# Changing Risk Behaviours and the HIV Epidemic: A Mathematical Analysis in the Context of Treatment as Prevention

**DOI:** 10.1371/journal.pone.0062321

**Published:** 2013-05-06

**Authors:** Bojan Ramadanovic, Krisztina Vasarhelyi, Ali Nadaf, Ralf W. Wittenberg, Julio S. G. Montaner, Evan Wood, Alexander R. Rutherford

**Affiliations:** 1 The IRMACS Centre, Simon Fraser University, Burnaby, British Columbia, Canada; 2 British Columbia Centre for Excellence in HIV/AIDS, Vancouver, British Columbia, Canada; 3 Faculty of Health Sciences, Simon Fraser University, Burnaby, British Columbia, Canada; 4 Department of Mathematics, Simon Fraser University, Burnaby, British Columbia, Canada; 5 Division of AIDS, Department of Medicine, Faculty of Medicine, University of British Columbia, Vancouver, British Columbia, Canada; Yale School of Public Health, United States of America

## Abstract

**Background:**

Expanding access to highly active antiretroviral therapy (HAART) has become an important approach to HIV prevention in recent years. Previous studies suggest that concomitant changes in risk behaviours may either help or hinder programs that use a Treatment as Prevention strategy.

**Analysis:**

We consider HIV-related risk behaviour as a social contagion in a deterministic compartmental model, which treats risk behaviour and HIV infection as linked processes, where acquiring risk behaviour is a prerequisite for contracting HIV. The equilibrium behaviour of the model is analysed to determine epidemic outcomes under conditions of expanding HAART coverage along with risk behaviours that change with HAART coverage. We determined the potential impact of changes in risk behaviour on the outcomes of Treatment as Prevention strategies. Model results show that HIV incidence and prevalence decline only above threshold levels of HAART coverage, which depends strongly on risk behaviour parameter values. Expanding HAART coverage with simultaneous reduction in risk behaviour act synergistically to accelerate the drop in HIV incidence and prevalence. Above the thresholds, additional HAART coverage is always sufficient to reverse the impact of HAART optimism on incidence and prevalence. Applying the model to an HIV epidemic in Vancouver, Canada, showed no evidence of HAART optimism in that setting.

**Conclusions:**

Our results suggest that Treatment as Prevention has significant potential for controlling the HIV epidemic once HAART coverage reaches a threshold. Furthermore, expanding HAART coverage combined with interventions targeting risk behaviours amplify the preventive impact, potentially driving the HIV epidemic to elimination.

## Introduction

Highly active antiretroviral therapy (HAART) suppresses HIV viral replication, which not only reduces morbidity and mortality [1], [2], but also the transmission of HIV [3]–[5]. As a result, HAART has emerged as a potentially high-impact global prevention strategy [6], [7]. Ecological and cohort studies have documented significant associations between increasing treatment coverage and declines in new HIV diagnoses [8] or incidence [9]. More recently, a randomized control trial found that HIV transmission in serodiscordant couples decreased by 96% when the infected partner received immediate HAART [10]. These findings are fuelling accelerated efforts to implement Treatment as Prevention programs worldwide, through expanding testing and offering earlier treatment to those infected with HIV. Implementation studies are either planned or currently underway throughout the world to evaluate the preventive effectiveness of Treatment as Prevention under field conditions [11].

Potential negative consequences of a large-scale expansion of treatment have been debated in the literature [6], [12]–[16]. One concern is the possible increase in risk behaviours over time [17], [18]. The argument is that expanding awareness of the beneficial effects of HAART can reduce fears of acquiring or transmitting HIV infection, which can lead to behavioural disinhibition commonly referred to as HAART optimism or risk compensation [4].

A number of empirical studies have investigated HAART optimism in heterosexual men and women, men who have sex with men (MSM), and injection drug users (IDU) [19]. The picture emerging from these studies is complex. Increases in risk behaviour since HAART was introduced in 1996 have been most often reported for MSM [20]–[33], but examples of no change [34] and also of decreasing risk behaviour have also been reported [35]. A number of studies of heterosexual individuals find that sexual risk behaviour drops or does not change [18], [21], [36], [37] after the initiation of HAART, but some report increasing risk behaviours [38]. For IDU, the evidence for HAART optimism affecting sexual and injection risk behaviour is also mixed, with studies reporting increased risk behaviour [39], decreased risk behaviour [40], and no change in risk behaviour [41].

The potential impact of HAART optimism on the population-level preventive effects of HAART have also been studied using mathematical models. For example, a compartmental model of the San Francisco MSM community incorporates the evolution of drug-resistant strains and assumes that sexual risk behaviour increases with time [42]. In this model, an only 10% increase in risk behaviour was sufficient to overwhelm the gains achieved in preventing new infections through expanded HAART coverage. However, this model assumes that drug resistance plays a significant role in curtailing benefits from expanded HAART coverage.

Several studies have concluded that HAART optimism can substantially limit or completely overwhelm the effectiveness of HAART in preventing new infections [43]–[46]. Other studies predicted benefits despite behavioural disinhibition [12], [47]. No clear pattern can be discerned from these studies. This may be due in part to variation in model assumptions. For example, studies which assume lower preventive efficacy for HAART, also tend to find greater negative impact due to HAART optimism. However, additional sources of variability may include the inherent heterogeneity in risk behaviour specific to geographical setting or risk group, as well as methodological differences between modelling studies.

One challenge is that the concept of HAART optimism is poorly defined [48], [49]. Changing social norms around HIV risk due to treatment can be envisaged to influence both HIV-positive and HIV-negative individuals because diffusion of opinions and attitudes affects the population as a whole. However, empirical studies of HAART optimism tend to focus on subgroups of those diagnosed with HIV or specifically those on treatment. Behavioural disinhibition in the undiagnosed or susceptible subpopulations may have equal or even greater influence on population-level preventive HAART effects [48], and at least one study of MSM in the Netherlands provides supporting evidence [32].

A methodological limitation common to many models of HAART optimism involves the representation of risk behaviour. As people respond to changing social norms by changing their attitudes and behaviours, they are also changing the social norms themselves. This is an example of a dynamic interaction that can have a profound systematic impact on the HIV epidemic. Mathematical models that treat HIV-related risk behaviour dynamically are rare [50]. In most models, risk behaviour is represented as an exogenous parameter, with a predefined value. Furthermore, these models typically make assumptions specific to one setting and, therefore, it may be difficult to translate their results to different situations and locations.

In this analysis, we examine in a general sense the role that either increasing or decreasing risk behaviours may play in influencing the population-level impact of Treatment as Prevention. Our approach is to represent the spread of risk behaviour as a social contagion [51], [52] and to use a two-disease model [53], [54] in which we treat both HIV and risk behaviour as infectious processes. In the context of HIV, two-disease models have been developed previously to study co-infections by HIV and TB [55]–[64] or HIV and gonorrhoea [65], [66]. We are not aware of other studies that applied this approach to HIV and risk behaviour.

In our model, both the acquisition of risk behaviour and infection with HIV are by contact with others. The acquisition of risk behaviour is a precondition for possible subsequent infection with HIV. We perform a mathematical analysis of the epidemiologically relevant equilibria for arbitrary parameter values. Detailed calculations and proofs are provided in the *Mathematical Supplement S1*. One benefit of this is that our results are quite general, not specific to any particular locale.

The model is used to investigate the impact on HIV incidence and prevalence of simultaneous changes in risk behaviour and HAART coverage. Risk behaviour may be influenced independently through targeted interventions such as harm reduction, or be coupled to HAART coverage through HAART optimism. Data on the HIV epidemic in Vancouver’s Downtown Eastside inner-city neighbourhood are used to demonstrate a specific application of the model.

## Analysis

### Model Description and Analysis of Model Behaviour

#### Model structure

**Figure 1:**
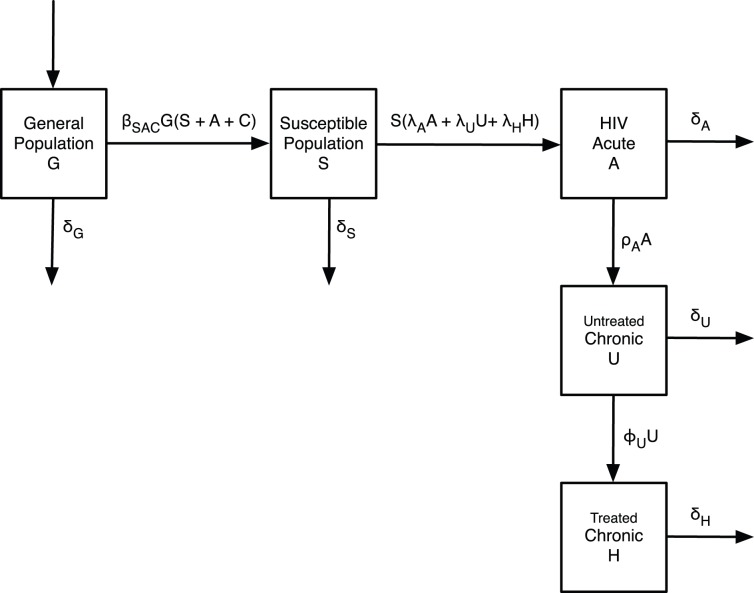
Compartmental model of the HIV epidemic linked to the spread of risk behaviour. The five compartments define states, which evolve with time according to the system of nonlinear ordinary differential equations (1).

Our deterministic compartmental model of an HIV epidemic driven by the spread of risk behaviour is illustrated in Figure 1. The model has five states or compartments consisting of the general subpopulation 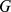 not engaging in risk behaviour, the susceptible subpopulation 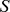 who engage in risk behaviour but are not infected by HIV, the HIV-positive subpopulation 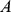 in the early acute phase of infection, the untreated HIV-positive subpopulation 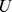 in the post-acute chronic phase of infection, and the HIV-positive subpopulation 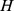 in the post-acute chronic phase who are receiving treatment with HAART. The chronic phase combines both the latent phase and AIDS. The mortality rate and infectiousness in the chronic phase are calculated as a time average of these values in each of the latent and AIDS phases. It is assumed that patients in the acute phase do not receive treatment.

The system of differential equations governing the time evolution of subpopulations in the model is
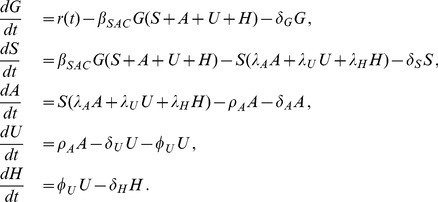
(1)


Individuals in the subpopulations 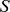 and 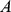, as well as in the two chronic phase subpopulations 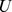 and 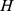, are assumed to equally influence individuals in the general subpopulation to engage in risk behaviour. This influence is modelled by a contact term with coefficient 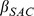. This coefficient may be interpreted as the probability per unit time that social influence between a single individual engaged in risk behaviour and one not engaged in risk behaviour will result in the latter individual becoming involved in risk behaviour. The infectivities of the subpopulations in the HIV acute phase, untreated HIV chronic phase, and treated HIV chronic phase are 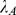, 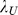, and 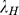, respectively. Specifically, 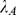 is the rate of infection per unit time between a single HIV-negative individual engaged in risk behaviour and an HIV-positive individual in the acute phase of infection. The infectivities 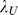 and 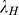 have analogous interpretations. Note that 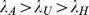, because HIV viral load is much higher in the acute phase than in the chronic phase of HIV infection and treatment further reduces viral load. The parameter 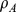 is the rate at which HIV-positive individuals transition from the acute phase to the chronic phase. In other words, 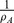 is the average duration of the acute phase. The parameter 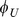 is the rate at which HIV chronic phase individuals are diagnosed and initiate treatment with HAART. The death rates in the 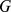, 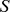, 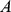, 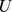, and 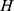 subpopulations are 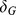, 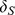, 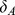, 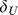, and 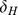, respectively. The term 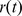 is the replenishment rate at which new individuals enter the model.

The infectivity parameters 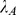, 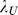, and 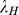 are influenced by factors such as the number of partners, length of partnerships, number of risk acts within a partnership, and the average probability of infection per single risk act between serodiscordant partners. This probability is in turn influenced by viral load. In this analysis, we primarily focus on the impact of risk behaviour on the infectivity parameters.

We assume that the death rates in the general subpopulation, susceptible subpopulation, acute phase HIV-positive subpopulation, and treated chronic phase population are equal and let 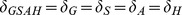. The death rate 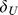 in the untreated chronic phase HIV-positive subpopulation takes into account transitions from the HIV latent phase to AIDS and subsequent death due to AIDS-related causes. As a result, we assume that 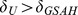. The death rate in the acute HIV phase is not elevated, because patients do not die of AIDS-related causes directly from the acute phase. The assumption that 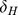 is equal to the death rate in the general population corresponds to assuming that patients on HAART who are virally suppressed have a normal life expectancy.

We make the simplifying assumption that the model population has constant size 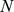, which entails setting 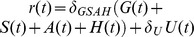. Substituting this expression for 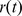 into the system of equations (1), dividing all equations by 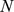, and rescaling the parameters gives the following system of equations:
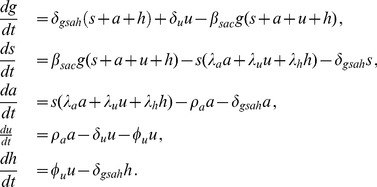
(2)


In these equations, 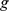, 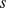, 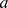, 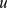, and 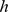 are the fractions of the population in each of the states 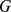, 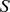, 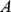, 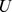, and 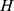, respectively. These functions satisfy the constraint that 
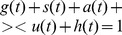
. Therefore, the system of equations (2) consists of only four independent equations. In order to understand the dynamics of the model defined by the system of equations (2), we study the equilibria of this system.

#### Model equilibria

The task of determining the model equilibria of the system of equations (2) can be simplified by combining the last two equations, which are linear equations with an inflow of 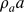. The total fraction of the population that is in the HIV chronic phase is 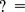 and the fraction of the HIV chronic phase population that is on treatment is 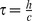. This gives the following system of equations for 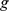, 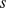, 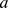, and 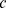:
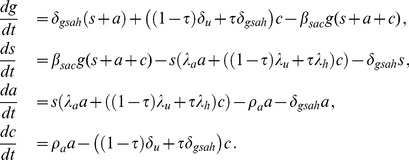
(3)


The last equation in the system (2) implies that at any equilibrium of this system, the equilibrium values 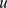 of 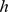 must satisfy
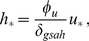
(4)where the subscript 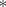 is used to denote a generic equilibrium value. Therefore, the value of 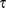 at any equilibrium for which 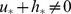 is



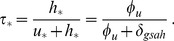
(5)This equation gives 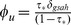. Therefore, we can replace the role of 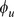 in the model by 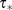, which is defined as the fraction of the HIV chronic phase subpopulation which is on treatment at equilibrium. This equilibrium HAART coverage parameter is treated as an exogenous parameter in the model.

To find the equilibria of the system of equations (3), it is sufficient to study the following system, which has 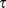 replaced by 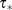:
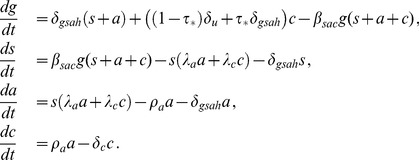
(6)


This system of equations has the same equilibria as (3). We have defined
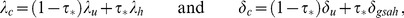
(7)which are the effective HIV chronic phase infectivity and death rate at equilibrium. These are obtained by using 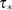 to calculate weighted averages from the infectivities and death rates in the undiagnosed and treated subpopulations. It follows from our assumptions 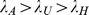 and 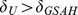 that the effective chronic phase infectivity and death rate in the equations (7) satisfy the inequalities 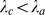 and 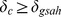.

The system of equations (6) is analysed rigorously in the *Mathematical Supplement S1* to this paper. It is found that if 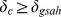 and 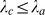, then the model exhibits three types of equilibrium states: the risk-free state in which there is neither endemic risk behaviour nor endemic HIV, the risk-endemic state in which risk behaviour is endemic but there is no endemic HIV, and the HIV-endemic state in which both risk behaviour and HIV are endemic.

Each of the equilibrium states may in turn be stable, depending on the values of the parameters in the model. The importance of stable equilibria is that they represent the state approached by the epidemic as time evolves. In the *Mathematical Supplement S1*, conditions for the stability of each of these three equilibria are determined and we shall simply quote the results here.

#### Risk-free equilibrium

The risk-free equilibrium state is stable if
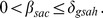
(8)


The population proportions at this equilibrium are
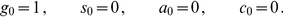
(9)


#### Risk-endemic equilibrium

The risk-endemic equilibrium is stable if
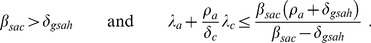
(10)


The population proportions at this equilibrium are
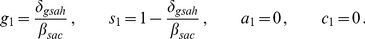
(11)


#### HIV-Endemic equilibrium

The HIV-endemic equilibrium is stable if
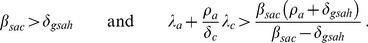
(12)


The population proportions at this equilibrium are
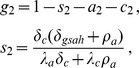


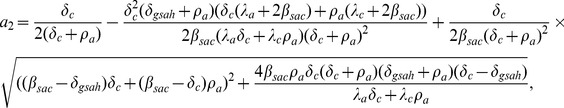
(13)

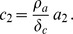



The interpretation of these results is that a minimum value for 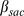, the rate of spread for risk behaviour, is required for risk behaviour to become endemic within the model. Given endemic risk behaviour, minimum values for the HIV infectivities 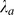 and 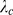 are required for HIV to become endemic in the model. Note that this minimum rate for the spread of HIV depends on the rate at which risk behaviour is spreading. The model describes two epidemics, with one driving the other.

Observe that for any given set of epidemiologically possible values of the parameters, exactly one of the three equilibria is stable. The behaviour of the system is determined by which equilibrium is stable. We can formulate public health goals in terms of guiding the system towards one of the equilibria by altering key parameters through interventions. For example, if our goal is the eradication of HIV, we need to determine what changes in parameter values would drive our system away from the HIV-endemic equilibrium by making it unstable. Alternatively, we may define a less ambitious goal of driving down HIV incidence and prevalence by changing the value of the HIV-endemic equilibrium in solution (13), through interventions designed to impact the parameters in the model.

#### Implementation of treatment

Treatment is implemented in the model by assuming that a specified proportion of the HIV-positive subpopulation in the chronic phase at equilibrium is receiving HAART, as was described in the previous subsection. Aside from the mathematical advantages of implementing treatment this way, using HAART coverage rather than treatment initiation rate 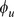 as a model parameter aligns the model better with standard public health metrics.

It is important to note that we define HAART coverage as the proportion of the entire chronically infected subpopulation – regardless of diagnostic status – that is on treatment with fully suppressed viral load. This definition does not address issues of low adherence, treatment failure and other factors that reduce effective coverage. In practice, HAART coverage is usually measured as a proportion of the diagnosed subpopulation prescribed HAART. This interpretation will lead to a higher HAART coverage value than the value in the model, because it uses the smaller reference population of only the diagnosed rather than all chronically infected individuals. Including treated patients with unsuppressed viral load would further increase the measured coverage above the HAART coverage value in the model. The analyses in this paper refer to the model definition of HAART coverage. Thus our estimates of the HAART coverage required to produce an effect on the course of the epidemic are likely to be lower than the actual coverage needed to achieve the same effect in practice.

We assume in this model that HIV-positive individuals in the acute phase are never on treatment, because it is unlikely that diagnosis and referral for treatment would be completed within the first two months of infection. This assumption is supported by data from British Columbia, Canada, where between 2006 and 2009 approximately 5% of newly diagnosed patients were in the acute phase [67]. Even fewer of these individuals would still be acutely infected when starting treatment.

We assume that individuals on HAART are not infectious and are not participating in disease dynamics [68], which implies that 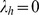. The fraction 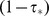 of the chronically infected subpopulation remains infectious in the model. This implies that HIV infectivity and death rate in the chronic phase from equations (7) simplify to
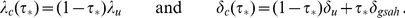
(14)


Both the stability condition of the HIV-endemic equilibrium in equations (12) and the population state at equilibrium in equations (13) depend on 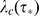 and 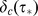. This implies that by changing HAART coverage, we can change both the HIV prevalence at equilibrium and the threshold for the switch from the state of endemic HIV to a state of endemic risk behaviour without HIV.

#### Estimation of model parameters

Model parameters were either taken directly from or were estimated using published sources. The ratio between infectivity in the acute phase and infectivity of untreated individuals in the chronic phase depends on the viral loads in the two disease phases. Therefore, it is convenient to write
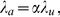
(15)where the ratio 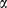 is a parameter related to the viral load in the two phases. We use 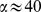 based on data from the Rakai, Uganda seroconversion study [69].

The length of the acute phase of an HIV infection is approximately 2 months. Therefore, we take 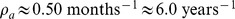. The life expectancy is defined as the average length of time from when individuals become susceptible to risk behaviour until death. The life expectancy for the uninfected subpopulation is taken to be 32 years, based on demographic information for Vancouver’s Downtown Eastside neighbourhood [68], which means that 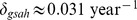. The life expectancy for an untreated HIV-positive individual is assumed to be 11 years [68], implying that 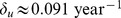. The values of these parameters are summarized in Table 1.

**Table 1:** Model parameters.

Parameter Description	Symbol	Model Analysis	DTES Value	Reference
progression rate from acute to chronic infection	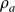	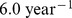	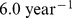	[78]
death rate for HIV-negative and acute phase HIV	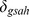	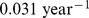	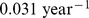	[68]
death rate for untreated chronic phase HIV	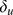	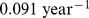	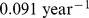	[68]
ratio of acute HIV infectivity to chronic infectivity	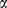	40	40	[69], [78]
risk behaviour propagation coefficient	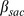	free	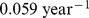	this paper
untreated chronic phase HIV infectivity	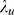	free	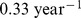	this paper
equilibrium HAART coverage for HIV chronic phase subpopulation	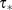	free	0.20	[68]

Values for the general model analysis and for the specific application to Vancouver’s Downtown Eastside are shown.

The parameters 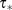, 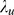, and 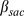 are treated as free parameters. The equilibrium HAART coverage 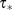 depends on the effectiveness of HIV testing programs and the guidelines for initiating treatment. The infectivity 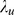 of untreated HIV-positive individuals depends on the level and nature of risk behaviour among HIV-positive individuals. Harm reduction programs would be expected to primarily impact 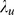. In this model, any impact on 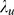 is also reflected in the acute phase infectivity, because 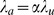. The risk behaviour propagation coefficient 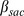 reflects the propensity for individuals to become engaged in risk behaviour and is modified by social influences such as HAART optimism.

### Model Results

#### Control and eradication of the HIV epidemic in the presence of risk behaviour

Risk behaviour is captured in the model through the parameters 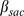 and 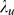. The parameter 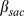 measures the rate of risk behaviour propagation and incorporates both the receptivity of susceptible individuals to becoming engaged in risk behaviour, and peer pressure that risk-engaged individuals exert, without making a distinction between the two effects. The infectivity parameter for untreated individuals in the chronic phase, 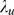, incorporates the frequency of risk behaviour, through the effect that risk behaviour has on infectivity. By equations (14) and (15), 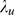 is related to both 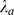 and 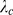. This determines the impact of risk behaviour on infectivity in both the acute and chronic phases in the model.

Analyses of equilibrium states, described in the *Model Equilibria* subsection, enable us to determine the necessary conditions for the persistence of the HIV epidemic, which occurs when the HIV-endemic equilibrium in equations (13) is stable. The stability condition for the HIV-endemic equilibrium in equation (12) can be rewritten using equations (14) and (15) as a condition on the undiagnosed chronic phase infectivity:
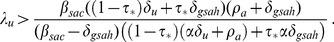
(16)


This condition for stability of the HIV-endemic equilibrium can be rewritten as the condition.
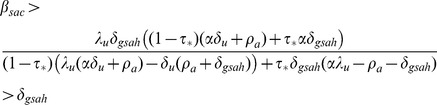
(17)


**Figure 2:**
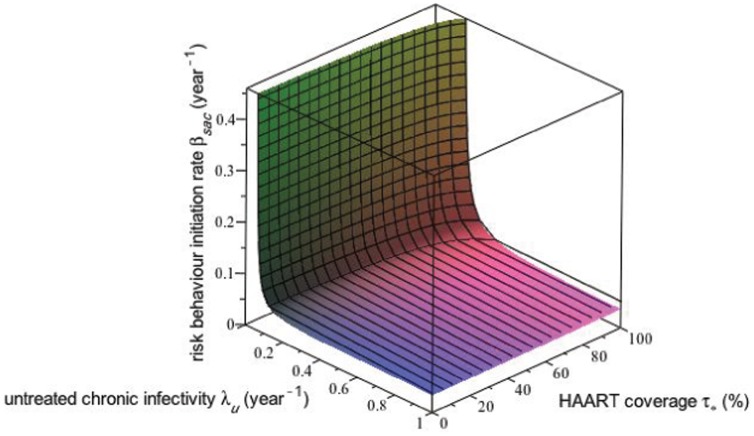
Surface representing the boundary that separates the HIV-endemic and HIV-free regions. The space is defined by risk behaviour propagation coefficient 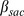, untreated chronic phase infectivity 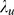, and equilibrium HAART coverage 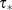. HIV is endemic above the plotted surface and extinct below it.

Substitution of 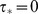 into equation (16) shows that if.
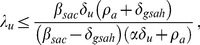
(18)then the HIV epidemic is not sustainable, even without HAART. Furthermore, if the risk-endemic equilibrium in equations (11) is stable, then risk behaviour persists even in the absence of an HIV epidemic. The maximum HAART coverage possible is 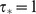. Substituting this into the inequality (16) shows that if
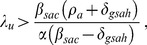
(19)then HAART cannot eliminate the HIV epidemic in the model. This occurs because individuals in the acute phase of HIV infection are never treated with HAART in the model and the inequality (19) is the condition for the epidemic to be sustainable with only transmissions from individuals in the acute phase. Provided that the HIV-endemic equilibrium is unstable when 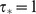 and stable when 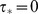, the condition on 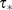 for the HIV-endemic equilibrium to be stable is



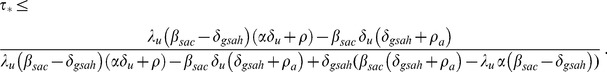
(20)The surface representing the boundary between regions where HIV is endemic and where it is extinct within the three-dimensional parameter space defined by 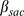, 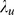, and 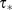 is obtained by setting 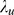 equal to the expression in (16). This surface is plotted in Figure 2, with the parameters 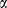, 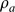, 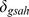, and 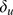 set equal to the values in Table 1. To make visualisation of these regions easier, we alternately fix one of the three parameters and plot the boundary curve in two dimensions.

**Figure 3:**
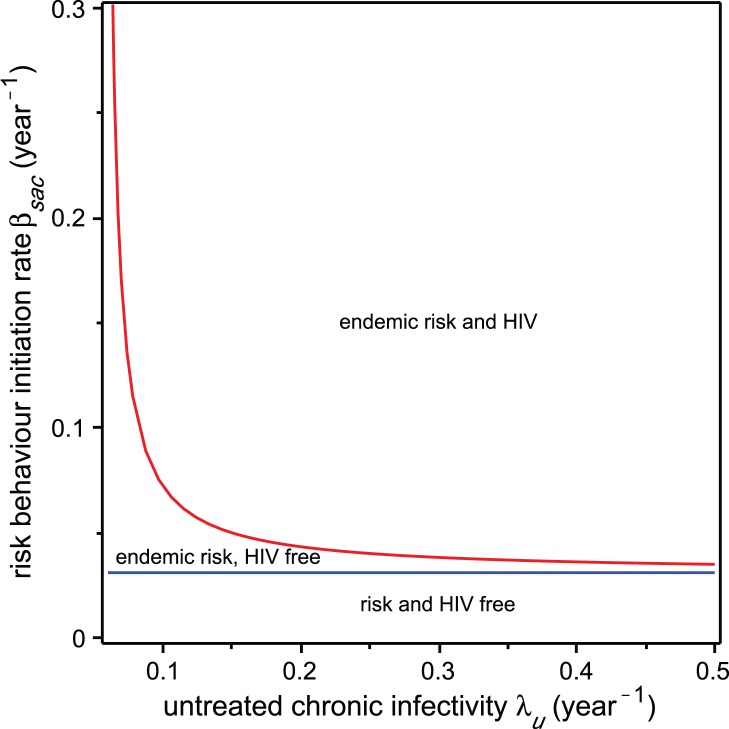
Equilibrium stability regions with no HAART coverage. Regions represent values of the risk behaviour propagation coefficient 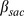 and the infectivity in the undiagnosed chronic HIV phase 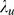 for which both HIV and risk behaviour are endemic, only risk behaviour is endemic, and neither are endemic. The red curve separates the HIV-endemic and HIV-free regions. The blue line separates risk behaviour-endemic and risk behaviour-free regions. The equilibrium HAART coverage parameter 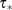 is set to 0 for this plot.

First, we set chronic phase HAART coverage 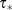 to 0 and use the inequalities (10) and (18) to plot regions for 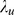 and 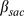 in the two-dimensional parameter space, where each of the risk-free, risk-endemic, and HIV-endemic equilibria are stable. The resulting diagram, known as a two-parameter bifurcation diagram, is shown in Figure 3. We find that the HIV epidemic can be eliminated by reducing either 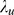 or 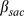 to a small enough value. The concave shape of the boundary curve implies that, at least theoretically, there exists an optimal strategy for driving the HIV epidemic to elimination. Starting with an HIV-endemic state, elimination of the HIV epidemic corresponds to driving the system along a path in the 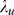 and 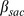 parameter space, which leaves the HIV-endemic region. The shortest such path is one possible definition of an optimal intervention. The concave curve implies that the shortest path to the boundary is neither vertical nor horizontal, regardless of where it starts within the HIV-endemic region. Therefore, a combination intervention, which impacts both 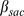 and 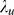, is optimal. Whether a theoretically optimal strategy is also optimal in practice depends on the cost or feasibility of the intervention. The model can be applied to real-world situations by modifying the path-length definition of optimality through weighting the 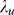 and 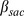 components of the path length by the cost associated with interventions that modify these parameters.

**Figure 4:**
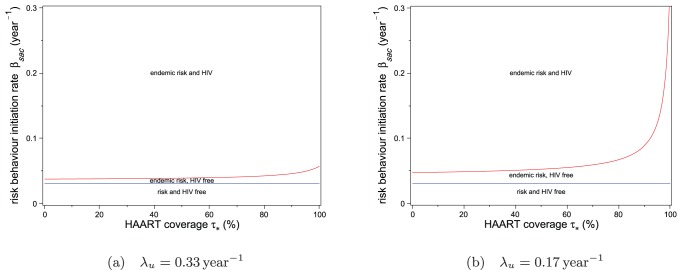
Equilibrium stability regions with fixed untreated chronic phase infectivity. The untreated chronic phase infectivity is fixed at (a) 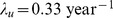 and (b) 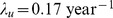 Regions represent values of the risk behaviour initiation rate 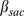 and equilibrium HAART coverage 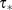 for which both HIV and risk behaviour are endemic, only risk behaviour is endemic, and neither HIV nor risk behaviour are endemic. The red curve separates the HIV-endemic and HIV-free regions. The blue line separates risk behaviour-endemic and risk behaviour-free regions.

Next, we show in Figure 4 the two-parameter bifurcation diagrams obtained by setting the value of 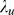 to 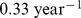 and to approximately half that value, 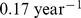. The stability regions in these bifurcation diagrams for 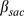 and 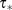 are obtained from the inequalities (10) and (17). The value of 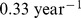 for 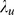 is the estimate obtained from our analysis of the Vancouver Downtown Eastside epidemic in the following section. As HAART coverage increases, the threshold value of 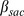 required for HIV to become endemic also increases. For 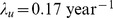, above 80% HAART coverage, the boundary curve rises rapidly, implying that by expanding HAART coverage, the epidemic can be eliminated even with rapid spread of risk behaviour. Similar to Figure 3, the boundary of the HIV-endemic region in Figure 4 is concave, implying that an optimal intervention involving 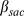 and 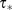 is a combination intervention which impacts both parameters.

**Figure 5:**
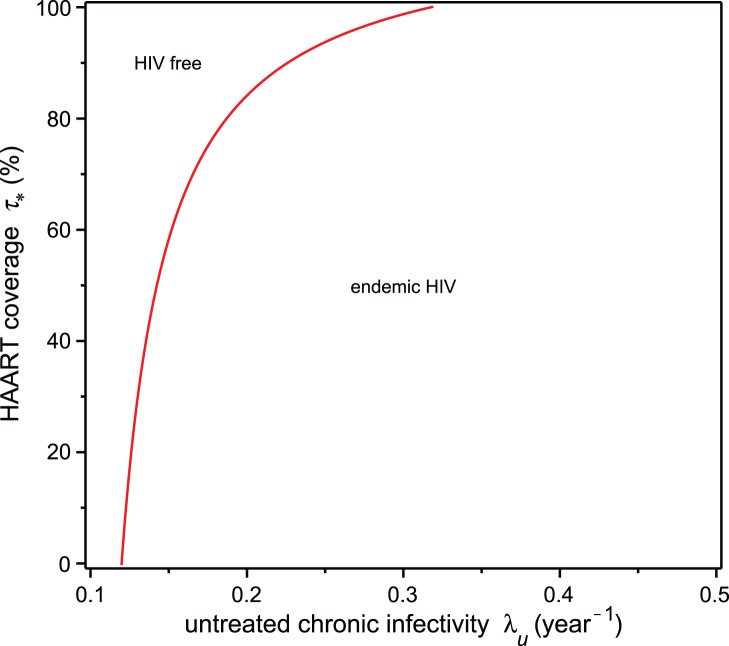
Equilibrium stability regions with fixed risk behaviour propagation rate. The red curve, which represents the equilibrium HAART coverage needed for extinction of the HIV epidemic as given by equation (20), separates the HIV-endemic and HIV-free regions. For 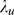 less than approximately 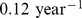, the HIV epidemic is not sustained. For 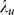 greater than approximately 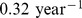, the epidemic cannot be eliminated by HAART in the chronic HIV phase alone. The risk behaviour propagation rate is fixed at 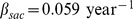.

We now fix the risk propagation coefficient 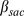 at 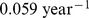, corresponding to the estimate used for the Vancouver Downtown Eastside analysis below and determine conditions on the infectivity 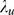 of untreated HIV-positive individuals and HAART coverage 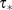 for endemic HIV. The stability regions in the two-parameter bifurcation diagram of 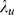 and 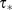 are shown in Figure 5. The graph in this figure is obtained from the inequality (20), with the parameter values taken from Table 1. It follows from equation (18) that if 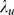 is less than approximately 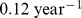, then an HIV epidemic is not sustainable, even without any HAART coverage. Equation (19) implies that if 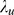 is greater than approximately 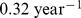, then endemic HIV would persist, even with complete HAART coverage. In our model, we are assuming that treatment with HAART only occurs in the chronic phase of the disease and in the last scenario the epidemic is being driven entirely by acute phase infections. From the graph in Figure 5, we can see that if 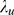 is less than approximately 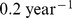, then eradication of the epidemic can be achieved with less than 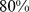 HAART coverage. Furthermore, for 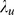 less than 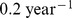, the level of HAART coverage required to eradicate the epidemic drops rapidly as 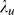 decreases.

Instead of eradicating the epidemic altogether, reducing HIV incidence and prevalence may be a more realistic short-term goal. Therefore, our next objective is to examine how equilibrium incidence and prevalence depend on the free parameters in the model. HIV prevalence, expressed as a proportion of the total population, is.
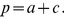
(21)


**Figure 6:**
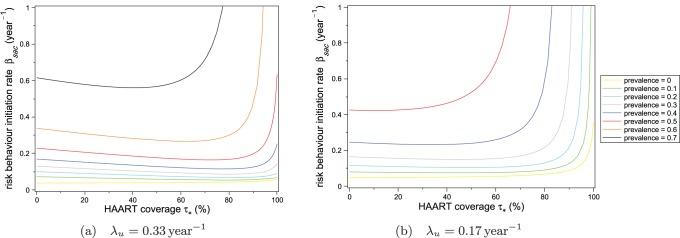
Level curves for HIV prevalence as a function of the risk behaviour propagation coefficient 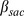 and equilibrium HAART coverage 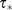. Equilibrium prevalence is shown as a fraction of the total population for two values of the untreated chronic phase infectivity: (a) 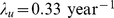 and (b) 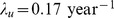. The level curves are curves along which prevalence is constant at the indicated value.

The equilibrium prevalence when HIV is endemic is.
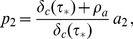
(22)where 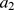 is given in equations (13) and 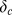 is given by equation (14). HIV incidence is defined as the number of new infections per unit time and given by
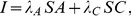
(23)where 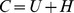 and 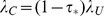. It is convenient to also express incidence relative to the size of the total population. Incidence per unit population is
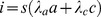
(24)and hence the incidence per unit population at the HIV-endemic equilibrium is



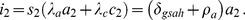
(25)
Figure 6 shows level curves for multiple values of equilibrium HIV prevalence, given by equation (21), as a function of 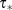 and 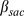, plotted for values of 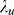 fixed at 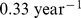 and 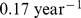. The value of 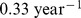 was chosen, because this is the estimate obtained for the Vancouver Downtown Eastside below. The prevalence level curves for 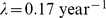 show the impact of reducing 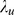 by approximately half. Each level curve represents the combination of the 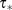 and 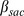 parameters which corresponds to constant HIV prevalence at the indicated value. Figure 6 shows that once HAART coverage increases beyond a threshold, the spread of risk behaviour has little further impact on HIV prevalence. Furthermore, this effect depends on 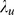, becoming more pronounced as infectivity drops. Reducing 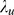 from 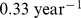 to 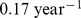 shifts the range in which HAART is highly effective at reducing prevalence from approximately 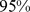 coverage to approximately 80% coverage.

The graphs in Figure 6 show that there is a maximum value of the risk propagation coefficient 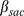 below which the HIV epidemic is not sustainable, because equilibrium prevalence is zero. This value can be computed using the inequality (17). When 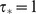, this inequality gives the following maximum value of 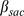 for which the epidemic can be eliminated through solely an increase in HAART coverage:
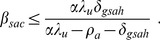
(26)


When 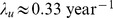, the maximum value of the risk propagation coefficient for which the HIV epidemic can be eliminated solely through HAART expansion is approximately 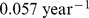. If 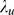 decreases to 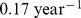, then this maximum value increases to 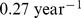.

These results show that expanding HAART coverage can be highly effective in containing and perhaps eradicating the HIV epidemic, provided that the infectivity of undiagnosed HIV-positive individuals is sufficiently low. The model further suggests that the benefit of using Treatment as Prevention increases significantly when combined with measures such as harm reduction that decrease untreated infectivity.

#### Impact of HAART optimism

HAART optimism is defined in this study as an increase in the social propagation of risk behaviour in an environment of expanded HAART coverage. It is incorporated in the model by assuming that the risk behaviour propagation coefficient 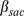 depends on the level of HAART coverage 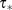. HAART optimism implies that 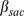 is an increasing function of 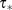 on the interval 0 to 1. Furthermore, 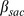 must be positive when 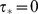, because people engage in risk behaviour even in the absence of HAART. For simplicity, we postulate that 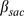 depends linearly on 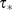 and write.
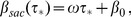
(27)where 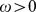 and 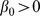. We refer to 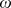 as the intensity of HAART optimism. The parameter 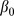 is the value of 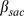 in the absence of HAART. We examine the impact of varying both 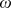 and 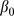 on HIV prevalence and incidence.

**Figure 7:**
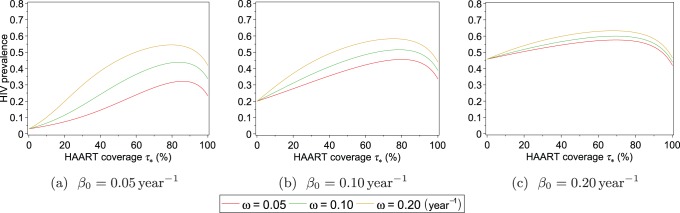
Equilibrium HIV prevalence as a function of equilibrium HAART coverage, with HAART optimism. Equilibrium HIV prevalence 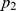, given by equation (22), is shown for three different values of the intensity of HAART optimism 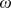 and the value of the infectivity in the absence of HAART optimism 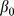. Prevalence is given as a fraction of the total population.



**Figure 8:**
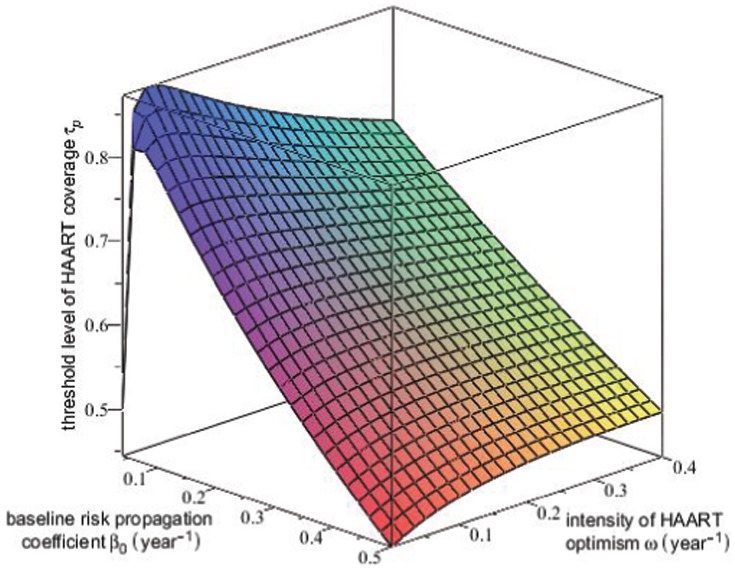
Threshold level of equilibrium HAART coverage for HIV prevalence. This surface plot shows the threshold level 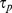 of equilibrium HAART coverage 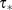 above which equilibrium HIV prevalence 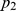 decreases with increasing HAART coverage, plotted as a function of 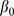, the value of the risk propagation coefficient in the absence of HAART, and the intensity of HAART optimism 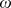. The rapid decrease in the 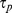 surface for small values of 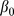 and 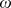 occurs because for these values of 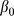 and 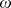 the HIV epidemic can be extinguished for large enough values of the equilibrium HAART coverage 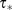.

Figure 7 shows equilibrium HIV prevalence 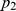 plotted as a function of HAART coverage 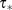 for three different values of 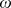 and 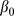. These plots are generated using equations (22) and (27), with the parameters 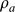, 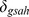, 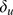, and 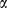 set to values given in Table 1 and 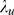 set to 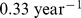. HAART optimism initially causes prevalence to increase. However, there is a threshold level of HAART coverage above which prevalence decreases, even in the presence of HAART optimism. As expected, larger values of the intensity of HAART optimism 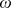 cause greater increases in prevalence for levels of HAART coverage below this threshold. This threshold value of 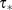 can be computed by numerically calculating the value of 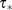 between 0 and 1 for which the derivative of equilibrium HIV prevalence 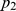 with respect to HAART coverage 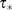,

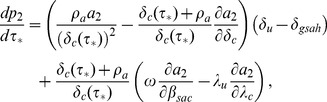
(28)is zero. We denote this threshold level of HAART coverage for prevalence by 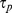. First we examine how 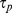 varies with 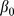. Setting the infectivity 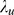 to 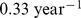 and 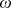 to 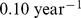, the threshold level of HAART coverage 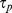 is approximately 0.84 when 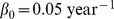 and it decreases to 0.69 when 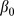 is increased to 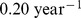. Now consider how 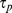 varies with 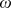. Setting 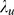 to 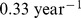 and 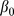 to 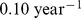, the value of 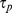 is 0.80 when 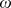 is 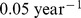 and it decreases to 0.75 when 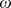 increases to 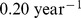. Not only does the threshold level of HAART coverage 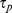 not increase with 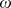, but it decreases slowly for this range of values for 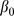 and 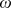. The dependence of 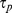 on 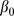 and 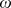 is shown in the surface plot in Figure 8.

**Figure 9:**
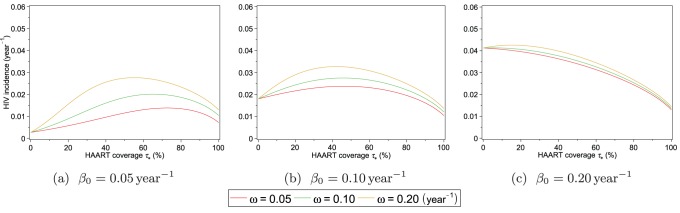
Equilibrium HIV incidence as a function of equilibrium HAART coverage, with HAART optimism. Equilibrium HIV incidence 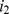, given by equation (25), is shown for three different values of the intensity of HAART optimism 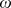 and the value of the infectivity in the absence of HAART optimism 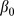.



**Figure 10:**
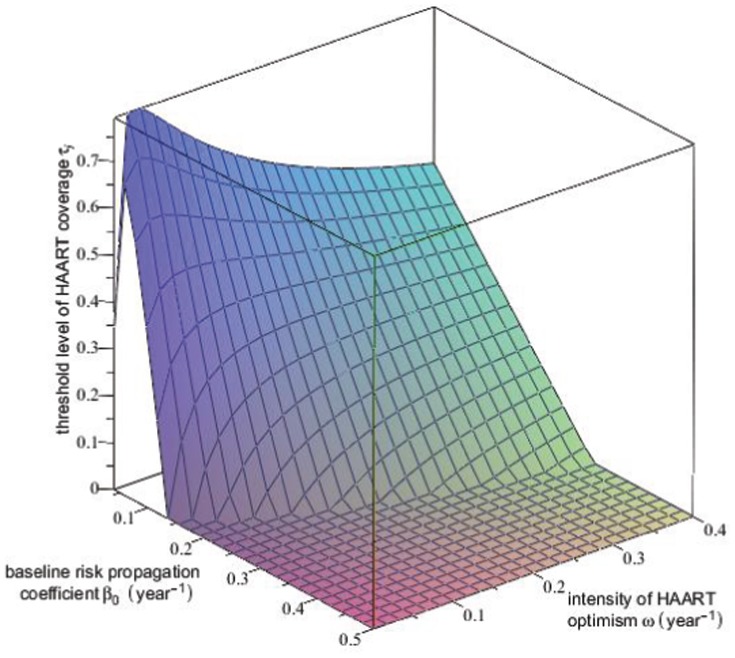
Threshold level of equilibrium HAART coverage for HIV incidence. This surface plot shows the threshold level 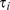 of equilibrium HAART coverage 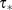 above which equilibrium HIV incidence 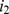 decreases with increasing HAART coverage, plotted as a function of 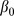, the value of the risk propagation coefficient in the absence of HAART, and the intensity of HAART optimism 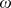. The rapid decrease in the 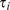 surface for small values of 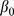 and 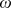 occurs because for these values of 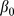 and 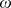 the HIV epidemic can be extinguished for large enough values of the equilibrium HAART coverage 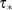. The region for which the 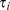 surface is identically 0 corresponds to 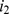 being a strictly decreasing function of 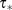 for 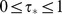.

Figure 9 shows equilibrium HIV incidence 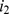 plotted as a function of HAART coverage 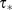 for three different values of 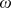 and 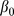. These plots are generated using equations (25) and (27), with the parameters 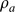, 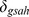, 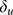, and 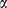 set to values given in Table 1 and 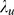 set to 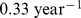. The plots of incidence in the presence of HAART optimism in Figure 8 show that for low levels of HAART coverage, HAART optimism causes incidence to increase. As with prevalence, there is a threshold level of HAART coverage, above which HAART causes incidence to decrease. The threshold level of HAART coverage for incidence is denoted by 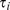. The value of 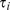 is computed by numerically solving for the value of 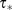 where.

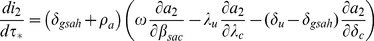
(29)is equal to zero. If 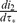 is never zero for 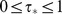, then it must be either strictly negative or strictly positive on this interval. If 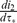 is strictly negative, then the threshold HAART coverage 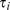 is 0 and if it is strictly positive, then 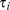 is 1. To illustrate how 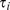 varies with 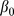 in Figures 9 and 10, we set 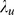 to 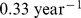 and 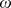 to 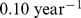. The threshold HAART coverage 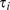 is then approximately 0.65 when 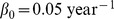; however, it decreases substantially to 0.047 when 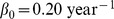. To examine how 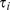 varies with 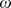, we set 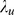 to 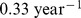 and 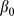 to 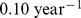. In this case, 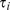 is approximately 0.46 and 0.42, when 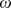 is 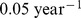 and 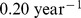, respectively. More generally, the dependence of the threshold HAART coverage 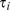 on 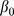 and 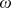 is shown as a surface plot in Figure 10. In this plot we can see that as 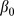 increases, eventually 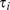 becomes zero. This occurs when 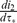 is strictly negative or in other words, 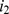 decreases with 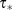 over the entire interval 0 to 1.

In summary, we find that in the presence of HAART optimism, HIV incidence and prevalence may either increase or decrease with increasing HAART coverage. For HIV prevalence, we demonstrated the existence of a threshold level of HAART coverage, below which prevalence increases with expanded HAART coverage, but above which HAART expansion overcomes the impact of HAART optimism and prevalence decreases with increasing HAART coverage. Likewise, we also demonstrated that there exists a threshold level of HAART coverage for HIV incidence. The threshold values for HAART coverage are different for prevalence and incidence. This difference stems from the fact that increasing HAART coverage reduces mortality among the HIV-positive population, which exerts upward pressure on prevalence, but not on incidence.

The relationship between the threshold levels for HAART coverage and the HAART optimism parameters is nonlinear and exhibits some counterintuitive properties. For example, we find that for some values of 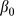 and 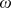, one or both of the threshold levels of HAART coverage may decrease slightly with increasing HAART optimism intensity 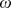. Of more significance, we find that the HAART coverage thresholds only increase significantly with HAART optimism intensity 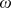 when both 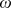 and the risk propagation coefficient 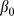 are very small. This scenario is unlikely to be epidemiologically relevant because at these values of the risk behaviour parameters the HIV epidemic is barely sustainable.

### The HIV Epidemic in Vancouver’s Downtown Eastside

In the 1990s, the inner-city Downtown Eastside neighbourhood of Vancouver experienced the most severe HIV epidemic in North America. HIV transmission was primarily driven by syringe sharing among injection drug users. Intensive harm reduction and efforts to expand HAART coverage were followed by a reduction in HIV incidence [9], [70]. However, HIV prevalence continues to be relatively high in the neighbourhood and HIV transmission has not been fully contained. We examine the combined HIV prevention impact of HAART and harm reduction, as well as the potential influence of HAART optimism in this setting.

#### Parameter values for the downtown eastside

The parameters 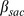, 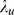, and 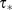, which were previously treated as free, are now set to values specific to the Downtown Eastside. The parameters 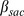 and 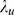 are influenced by complex behavioural factors that are difficult to measure. However, equations (13) link parameters to measurable demographic properties of the epidemic, such as the fraction of the population that is infected with HIV and the fraction of the population that is engaged in risk behaviour. Demographic data for the Downtown Eastside are available for 1999 [68]. This study reports that the size of the Downtown Eastside community was 19,815 individuals and that there was no significant net migration of HIV-positive and HIV-negative individuals. Furthermore, HIV prevalence was approximately 7%, approximately 20% of HIV-positive individuals were receiving HAART, and an estimated 5000 injection drug users were engaged in risk behaviour [68]. By assuming that the HIV epidemic in the Downtown Eastside is reasonably close to equilibrium, then we can use results from the study [68] to solve for 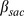, 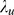, and 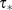.

First we solve for 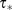. At the HIV endemic equilibrium in equations (13), the fraction of the total HIV-positive population that is on HAART is
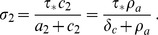
(30)


Substituting for 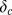 from equation (7) and solving for 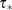 gives
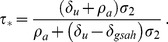
(31)


Therefore, using 0.20 as the estimate for 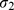 and substituting values for the other parameters from Table 1 gives approximately 0.20 for 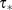.

To solve for 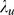, we use the equation for 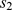 in (13), along with the equations (14) and (15). The result is that
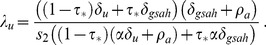
(32)


The study in reference [68] estimates 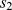, the fraction of the population that is engaged in injection drug use risk behaviour, but is HIV-negative, as 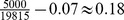. Substituting this value for 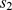 into equation (32), along with 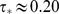 and the values of the other parameters taken from Table 1 gives 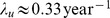. Harm reduction efforts in the Downtown Eastside have likely reduced 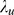 significantly from its 1999 value. A drop in syringe sharing behaviour by a factor of approximately three between 1999 and 2007 has been reported [71]. We explore the impact of this reduction in the subsection below.

The parameter 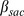 is obtained by setting HIV prevalence 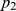 from equation (22) to 0.07 and solving for 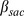. The result is 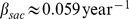. The Downtown Eastside values for all parameters in the model are listed in Table 1.

#### Evaluating the impact of harm reduction and HAART

Harm reduction programs have the effect of reducing 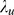, the infectivity of undiagnosed HIV-positive individuals. We examine the impact of expanded HAART coverage on equilibrium HIV prevalence and incidence post-1999 in the Downtown Eastside. All parameters other than 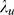 are set equal to their Downtown Eastside values in Table 1. The risk propagation coefficient 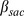 is fixed at 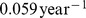 and does not depend on HAART coverage 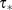 in this subsection.

**Figure 11:**
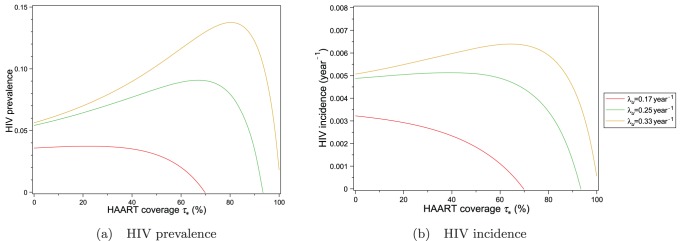
HIV prevalence and incidence in Vancouver’s Downtown Eastside as a function of equilibrium HAART coverage for different values of untreated HIV chronic phase infectivity. The impact of equilibrium HAART coverage 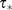 on HIV prevalence and incidence is shown for different values of the untreated chronic phase infectivity 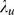. These graphs illustrate the potential impact of reducing 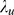 through harm reduction programs. For these plots, it is assumed that there is no HAART optimism.

The value of 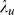 in the Downtown Eastside is likely to have decreased since 1999 as a result of harm reduction. The graphs in Figure 11 show the impact on HIV incidence and prevalence of reducing 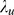 from 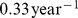 to 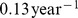 in decrements of 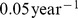. When 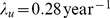, elimination of the epidemic is theoretically possible by expansion of HAART to 100% coverage of the population in the chronic phase of HIV infection. The inequality (19) gives the threshold value of 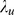, above which the epidemic cannot be eliminated solely by increasing HAART coverage for individuals in the HIV chronic phase. In the absence of HAART optimism, this threshold value for 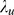 in the Downtown Eastside is approximately 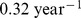. Further reduction of 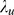 beyond this threshold improves the effectiveness of HAART expansion in containing the epidemic. For example, if 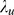 were 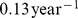 in the Downtown Eastside, then both equilibrium prevalence and incidence would decrease significantly when HAART coverage exceeds 50% and equilibrium prevalence would be reduced to zero when HAART coverage exceeds approximately 65%. These results highlight the importance of combining harm reduction and HAART expansion to reduce HIV incidence and prevalence.

#### HAART optimism in the downtown eastside

**Figure 12:**
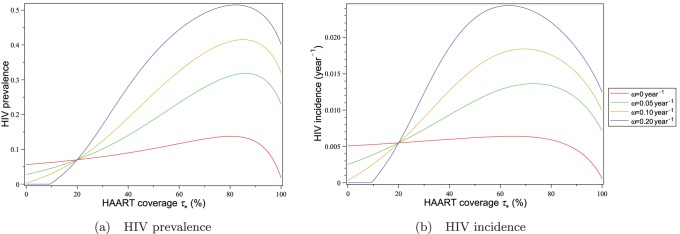
HIV prevalence and incidence in Vancouver’s Downtown Eastside for different levels of HAART optimism. The impact of equilibrium HAART coverage 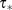 on HIV prevalence and incidence is shown for different values of the intensity of HAART optimism 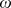. For each curve in these plots, the value of 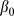 is chosen such that 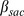 is equal to 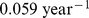 when 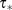 is 0.20.

HAART optimism in the Downtown Eastside is modelled by assuming that the risk propagation coefficient 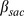 depends on HAART coverage according to equation (27). For different values of the intensity of HAART optimism 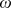, the resulting dependence of HIV incidence and prevalence on HAART coverage are plotted in Figure 12. Parameter values for these plots are taken from the Downtown Eastside values in Table 1. For each value of 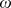 in these plots, the value of 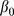 is chosen so that 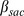 is equal to 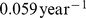 when 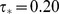.

The graphs in Figure 12 show that in 1999 HAART optimism would have caused both HIV incidence and prevalence in the Downtown Eastside to increase significantly. The reason for this is that the relatively high untreated HIV chronic phase infectivity 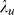 would have caused any increase in risk behaviour to drive up HIV incidence and prevalence significantly. However, for incidence, there is no empirical evidence for such an increase after HAART was introduced, on the contrary, incidence was found to decrease [9], [70]. This implies that if HAART optimism existed at all, it could have played only a minor role in the HIV epidemic in Vancouver’s Downtown Eastside.

## Discussion

### Main Findings

Using a mathematical model for the spread of HIV driven by risk behaviour as a social contagion, we mapped possible outcomes for the HIV epidemic in the context of expanding HAART coverage. We focused specifically on: (1) the potential for Treatment as Prevention to control the HIV epidemic in the context of changing risk behaviours; (2) the combined effects of HAART expansion and interventions targeting risk behaviours; and (3) the impact of HAART optimism.

Our results show that Treatment as Prevention has the potential to be a powerful strategy for controlling the spread of HIV. We find that comprehensive programs securing high HAART coverage could substantially reduce HIV incidence and potentially eliminate the epidemic. While returns on prioritizing high HAART coverage are likely to be great, they do not increase linearly and are influenced by the prevailing risk conditions. Consequently when high HAART coverage is supported by interventions targeting risk behaviour, these interventions work synergistically to drive the epidemic to lower levels of equilibrium incidence and prevalence. Benefits of interventions accelerate as we approach elimination of the HIV epidemic.

It has been argued that gains due to increased HAART coverage could be undermined by an increase in risk behaviour induced by HAART optimism [4], [17], [18]. Is this inevitable and if not, under what circumstances does it occur?

The dynamic representation of risk behaviour in our model enables us to take a unique approach to examining the potential epidemiological impact of HAART optimism in the population as a whole. In our model, the spread of risk behaviour in the community has two components: a baseline risk behaviour unrelated to HAART coverage and an additional risk behaviour arising from the intensity of HAART optimism. For low levels of HAART coverage, we find that the preventive benefits of HAART can indeed be overwhelmed by the negative impact of HAART optimism, leading to an increase in equilibrium HIV incidence and prevalence. However, typically there exists a threshold above which the preventive benefits of HAART gain the upper hand and further increases in HAART coverage reduce equilibrium incidence. Likewise, there exists a similar HAART coverage threshold for equilibrium prevalence. These threshold HAART coverage levels have important public health implications.

It is more informative to draw conclusions from HIV incidence, because the effect of HAART on prevalence is confounded by its impact on the life expectancy of treated patients. Equilibrium HIV incidence declines for increasing HAART coverage above the threshold. For high values of the baseline risk behaviour 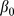, the threshold is constantly zero. In this case, expansion of HAART will consistently yield public health benefits, regardless of the presence of HAART optimism. For lower values of the baseline risk behaviour, the threshold increases initially with the intensity of HAART optimism, but ultimately levels at a value no higher than approximately 65%. Although for this case low values of HAART coverage can cause an increase in incidence, it should be possible to overcome the effects of HAART optimism at attainable levels of HAART coverage.

The Vancouver Downtown Eastside is an example of a setting, which today can be considered an environment with relatively low levels of risk behaviour propagation according to our definition. Intensive harm reduction beginning in the early 1990s resulted in a major shift in injection behaviours in this neighbourhood. Most IDU are now aware of the risks of HIV infection and avoid sharing syringes [72]. Applying our model to this setting, we showed that HIV incidence and prevalence would have had to increase substantially with behavioural disinhibition if HAART optimism existed in the community. This has not been observed empirically. In fact, HIV incidence has decreased over time [9] and a recent study found no evidence of HAART optimism in the Downtown Eastside [73]. Whether or not social norms operate to limit the resurgence of risk behaviours in environments with low levels of risk behaviour propagation, such as the Downtown Eastside, HAART optimism may exist in this context and addressing risk behaviours is likely to be important.

Our results also suggest that the potential HIV prevention benefits of HAART in high-risk environments could be substantial, despite any behavioural shifts due to HAART optimism and this could be a factor to consider in planning interventions. Sufficiently aggressive expansion of HAART access in these settings would reduce incidence. Furthermore, additional harm reduction, behavioural or substance use interventions are expected to boost and stabilize these effects. This could have relevance for some IDU and MSM groups. HIV infection rates among IDU in parts of Eastern Europe and Central Asia continue to be very high today due to high rates of syringe sharing and unprotected sex [74]. Containing the HIV epidemic in MSM groups where risk behaviours continue to be frequent, remains a challenge and resurgent epidemics are seen [32], [75]. Our model suggests that achieving and maintaining high HAART coverage in these types of settings could have major benefits in reducing incidence, especially in combination prevention strategies. HAART optimism is not likely to substantially diminish these benefits.

### Strengths and Limitations

One of the strengths of our method is that we are able to draw broadly valid qualitative conclusions about the epidemiological effects of changing risk behaviours. This has been possible because the model is simple enough to be mathematically tractable, which enabled us to prove the existence and describe the nature of all equilibria. Another strength of our approach is that we did not restrict much of our analysis to parameter values for the HIV epidemic in any specific setting, but examined model behaviour over the entire parameter space.

Through linking risk behaviour dynamically to HIV transmission, we were able to incorporate aspects of social influence in the production of HIV risk into the model. Representing risk behaviour as a social contagion has been used for modelling health issues such as obesity, smoking, drug use, and alcohol consumption [76]. The dynamic risk behaviour component of the model allowed us to study the effects of HAART optimism on the HIV epidemic in a novel way. Our analysis of risk behaviour encompasses the wider population, including both HIV negative and HIV infected subpopulations.

Although a strength, the generality of our analysis is also a limitation because it forces us to focus on the equilibrium states of the model. Non-equilibrium analysis can provide useful information on the short-term response of the epidemic to interventions. However, this type of analysis must consider a specific setting, because knowledge of the current state of the epidemic is necessary to generate reliable projections.

Although risk behaviour interacts with other components of the model dynamically, our treatment of it is still quite simplistic. Propagation of risk behaviour is assumed to occur only through peer interaction. Moreover, we have assumed that different types of risk behaviour and their ensuing transmission channels can be aggregated into a single generic risk behaviour and transmission channel. Furthermore, once risk behaviour is initiated, it can never stop in the model, making our predictions more pessimistic than might be the case with more realistic assumptions about risk behaviours.

HAART optimism is incorporated into the model by assuming that higher levels of HAART coverage lead to an increase in the risk behaviour propagation rate. In the absence of data, we assume a simple linear relationship between these two parameters. Even with a more general dependence of the risk behaviour propagation rate on HAART coverage, a linear relationship would still be a good approximation as long as there are no large changes in HAART coverage. Mathematically, this means that the linear relationship corresponds to the first two terms in a Taylor series expansion of the risk behaviour propagation rate as a function of HAART coverage.

We neglected drug resistance to retain model simplicity. This assumption is supported by findings in British Columbia where rates of acquired and primary resistance have been declining since 1996, despite marked expansion of HAART [77]. Due to this assumption, our conclusions should be interpreted with caution in the context of settings where drug resistance is an important problem. Other similifications that we made include omitting details on risk groups, variability in risk behaviours, social network features, testing strategies and combining the latent and AIDS stages of infection into a single chronic infection phase.

## Conclusions

Our analysis suggests that Treatment as Prevention has significant potential for containing the the HIV epidemic. However, substantial gains in reducing HIV incidence and prevalence are only achieved at or near critical coverage levels for HAART or other interventions. Therefore, determining critical HAART coverage levels may help develop more effective Treatment as Prevention programs. Prioritizing sufficiently high HAART coverage and incorporating interventions to reduce risk behaviour will amplify the preventive impact, possibly even eliminating the HIV epidemic. Local epidemiological conditions play a strong role in how much HAART coverage is required for controlling the HIV epidemic. The requirement for sufficiently high HAART coverage may extend to undiagnosed infections, necessitating comprehensive testing to be a part of a total prevention package. While HAART optimism promotes the growth of the HIV epidemic, this can be controlled by increasing HAART coverage and behavioural interventions. Therefore, our findings do not confirm previously predicted overwhelming negative impacts of HAART optimism on the benefits of Treatment as Prevention. In conclusion, an important public health lesson that emerged from this modelling exercise is that with Treatment as Prevention, half measures do not necessarily mean half results, while full commitment to comprehensive programs has substantial potential for controlling the HIV epidemic.

## Supporting Information

Mathematical Supplement S1 (PDF)
