# Supplementary material for: Changing Risk Behaviours and the HIV Epidemic: A Mathematical Analysis in the Context of Treatment as Prevention
Source: PLoS One. 2013 May 6;8(5):e62321. doi: 10.1371/journal.pone.0062321 (PMC3646049; doi:10.1371/journal.pone.0062321)
Supplement: Mathematical Supplement S1 — (PDF) [file pone.0062321.s001.pdf]

# S1: Mathematical Supplement

## *Changing Risk Behaviours and the HIV Epidemic: A Mathematical Analysis in the Context of Treatment as Prevention*

A. R. Rutherford
The IRMACS Centre and Department of Mathematics
Simon Fraser University
Burnaby, British Columbia, Canada
sandyr@irmacs.sfu.ca

In these supplementary notes, we prove a number of results on the equilibrium points and stability of the system of differential equations which define the risk-hiv model in the main paper. It is assumed that the reader is familiar with the methods and language of dynamical systems. For readers interested in an introduction to the field, we suggest [1].

## 1 The Model

The model describes a two disease system in which becoming infected with the first disease is a prerequisite for becoming susceptible to the second disease. The first disease represents risk behaviour, which spreads in the population through social influence. The second disease is HIV, which requires existence of risk behaviour to propagate. HIV is modelled as a two stage, with the first called the acute phase and the second the chronic phase.

We define the model so that the total population in the system is constant. The fraction of the general population not engaged in risk behaviour is denoted by $g$. The fraction of the population engaged in risk behaviour and susceptible to HIV is denoted by $s$. The fractions of the population in the acute and chronic HIV phases are denoted by $a$ and $c$, respectively. Individuals in the $s$, $a$, and $c$ groups influence members in the general population to engage in risk behaviour at a rate per person $\beta_{sac}$. The subpopulations $g$, $s$, and $a$ have the same death rate $\delta_{gsa}$. The death rate of the HIV chronic phase is $\delta_c$. The rate per person that HIV positive individuals move from the acute phase to the chronic phase is $\rho_a$. The infectivity of HIV positive individuals in the acute and chronic phases are $\lambda_a$ and $\lambda_c$, respectively. The model is described by the following system of four differential equations, which correspond to the system of equations (6) in the main paper, with $\delta_{gsah}$ replaced by $\delta_{gsa}$ and equation (7) in the main paper used to define $\lambda_c$ and $\delta_c$:

$$\begin{aligned}
\frac{dg}{dt} &= \delta_{gsa}\,(s+a) + \delta_c\,c - \beta_{sac}\,g(s+a+c)\,,\\
\frac{ds}{dt} &= \beta_{sac}\,g(s+a+c) - \lambda_a\,sa - \lambda_c\,sc - \delta_{gsa}\,s\,,\\
\frac{da}{dt} &= \lambda_a sa + \lambda_l sc - \rho_a a - \delta_{gsa} a\\
\frac{dc}{dt} &= \rho_a a - \delta_c c\,.
\end{aligned} \tag{S1.1}$$

We rescale to dimensionless time by multiplying $t$ by $\delta_{gsa}$. Relabelling the parameters to reflect these rescalings, as well as writing $\phi = \frac{\lambda_c}{\lambda_a}$ gives the system of equations,

$$\begin{aligned}\frac{dg}{dt} &= s + a + \delta c - \beta g(s + a + c)\,,\\ \frac{ds}{dt} &= \beta g(s + a + c) - \lambda s(a + \phi c) - s\,,\\ \frac{da}{dt} &= \lambda s(a + \phi c) - (\rho + 1)a\,,\\ \frac{dc}{dt} &= \rho a - \delta c\,.\end{aligned} \tag{S1.2}$$

The parameters $\beta$, $\lambda$, and $\delta$ are all assumed to be strictly positive. The parameter $\phi$ satisfies $0 < \phi < 1$. The physically relevant region for this system of equations is the simplex

$$\mathcal{R} = \{(g, s, a, c) \mid g \geq 0,\ s \geq 0,\ a \geq 0,\ c \geq 0,\ g + s + a + c = 1\}\ . \tag{S1.3}$$

The variables $g$, $s$, $a$, and $c$ satisfy the constraint $g + s + a + c = 1$, which allows us to reduce the system of four equation (S1.2) to the following three equations:

$$\begin{aligned}\frac{ds}{dt} &= \beta(1 - s - a - l)(s + a + l) - \lambda s(a + \phi l) - s\,,\\ \frac{da}{dt} &= \lambda s(a + \phi l) - (\rho + 1)a\,,\\ \frac{dc}{dt} &= \rho a - \delta l\,.\end{aligned} \tag{S1.4}$$

The physically relevant region for this reduced system of equations is the convex hull

$$\mathcal{R}_1 = \{(s, a, c) \mid s \geq 0,\ a \geq 0,\ c \geq 0,\ s + a + c \leq 1\}\ . \tag{S1.5}$$

To show that the system of equations (S1.4) is a well-defined model, it is necessary to verify that $\mathcal{R}_1$ is a trapping region. To prove this note that the vector field defined by (S1.4) is inward pointing at every point on the boundary of $\mathcal{R}_1$, except at $(s, a, c) = (0, 0, 0)$, which is an equilibrium point.

## 2 Equilibrium Points

To find the equilibrium points of the system of equations (S1.4), set $\frac{ds}{dt} = 0$, $\frac{da}{dt} = 0$, and $\frac{dc}{dt} = 0$ and find all real roots of the resulting algebraic system.

The risk-free equilibrium point

$$s_0 = 0\,, \qquad a_0 = 0\,, \qquad c_0 = 0 \tag{S1.6}$$

of the system (S1.4) corresponds to no risk behaviour, nor any disease in the population.

The second equilibrium point corresponds to endemic risk behaviour, but no disease in the system. The value of this equilibrium point, which we call the risk-endemic equilibrium point, is

$$s_1 = 1 - \frac{1}{\beta} \qquad a_1 = 0\,, \qquad c_1 = 0\,. \tag{S1.7}$$

Observe that the risk-endemic equilibrium point is in the physical region $\mathcal{R}_1$ if $\beta \geq 1$.

To find the remaining equilibrium points, assume that $s > 0$, $a > 0$, and $c > 0$ in the system of equations

$$0 = \beta(1 - s - a - c)(s + a + c) - \lambda s(a + \phi c) - s\,, \tag{S1.8a}$$

$$0 = \lambda s(a + \phi c) - (\rho + 1)a\,, \tag{S1.8b}$$

$$0 = \rho a - \delta c\,. \tag{S1.8c}$$

These equilibrium points would correspond to both endemic risk behaviour and endemic disease. Equation (S1.8c) gives

$$c = \frac{\gamma}{\delta} a\,. \tag{S1.9}$$

Substituting this into equation (S1.8b) gives

$$s = \frac{\delta(1 + \rho)}{\lambda(\delta + \phi\rho)}\,. \tag{S1.10}$$

Substituting from equations (S1.9) and (S1.10) into equation (S1.8a) gives

$$\beta\left(1 - \frac{\delta(1 + \rho)}{\lambda(\delta + \phi\rho)} - \left(1 + \frac{\rho}{\delta}\right)a\right)\left(\frac{\delta(1 + \rho)}{\lambda(\delta + \phi\rho)} + \left(1 + \frac{\rho}{\delta}\right)a\right) - \lambda\left(\frac{\delta(1 + \rho)}{\lambda(\delta + \phi\rho)}\right)\left(1 + \frac{\phi\rho}{\delta}\right)a - \frac{\delta(1 + \rho)}{\lambda(\delta + \phi\rho)} = 0\,. \tag{S1.11}$$

The quadratic equation (S1.11) may have zero, one or two real roots, which implies that the system may have as many as two equilibrium points at which the disease is endemic. However, it is shown below in Lemma 1 that if $\delta \geq 1$, then there can be at most one disease-endemic equilibrium point in the region $\mathcal{R}$. In terms of the original model equations (S1.1) this implies that if $\delta_c \geq \delta_{gas}$, then there is at most one endemic HIV equilibrium point. Deaths caused by HIV occur after the acute phase, so this assumption corresponds to assuming that HIV infection increases the death rate.

The first step in proving that $\delta \geq 1$ implies that there can be at most one disease-endemic equilibrium point is to simplify equation (S1.11) through the change in parameters

$$\begin{aligned} \psi_1 &= 1 + \frac{\rho}{\delta}\,, \\ \psi_2 &= 1 + \frac{\phi\rho}{\delta}\,, \\ \psi_3 &= \frac{\delta(1 + \rho)}{\delta + \phi\rho}\,. \end{aligned} \tag{S1.12}$$

Note that the map

$$g : (\rho, \delta, \phi) \mapsto (\psi_1, \psi_2, \psi_3) \tag{S1.13}$$

is a diffeomorphism of

$$\Omega_1 = \{(\rho, \delta, \phi) \mid \rho > 0,\ \delta > 0,\ \phi \geq 0\} \tag{S1.14}$$

to

$$\Omega_2 = \{(\psi_1, \psi_2, \psi_3) \mid \psi_1 > 1,\ \psi_2 > 1,\ \psi_3 > 0\}\,. \tag{S1.15}$$

The inverse is

$$g^{-1}(\psi_1, \psi_2, \psi_3) = \left(\psi_2\psi_3 - 1, \frac{\psi_2\psi_3 - 1}{\psi_1 - 1}, \frac{\psi_2 - 1}{\psi_1 - 1}\right)\,. \tag{S1.16}$$

In terms of the new parameters, the quadratic equation (S1.11) becomes

$$\beta\lambda^2\psi_1^2 a^2 - \left(\beta\left(\lambda^2\psi_1 - 2\lambda\psi_1\psi_3\right) - \lambda^2\psi_2\psi_3\right)a + (1 - \beta)\lambda\psi_3 + \beta\psi_3^2 = 0 \tag{S1.17}$$

and the condition $\delta \geq 1$ is equivalent to $\psi_2\psi_3 \geq \psi_1$. The result that there can be at most one disease-endemic equilibrium point follows from the following lemma.

**Lemma 1.** *If $\psi_2\psi_3 \geq \psi_1$, then the equation (S1.17) has at most one positive root.*

*Proof.* For the equation (S1.17) to have two positive roots, the coefficient of $a$ must be strictly negative, which is equivalent to

$$\lambda\left(\beta\psi_1 - \psi_2\psi_3\right) - 2\beta\psi_1\psi_3 > 0\,. \tag{S1.18}$$

This implies that

$$\lambda(\beta\psi_1 - \psi_1) - 2\beta\psi_1\psi_3 > 0\,, \tag{S1.19}$$

because $\psi_2\psi_3 \geq \psi_1$. The inequality (S1.19) simplifies to

$$\psi_3 < \frac{\lambda(\beta - 1)}{2\beta}\,. \tag{S1.20}$$

For equation (S1.17) to have two roots greater than or equal to zero, the constant term must satisfy

$$\psi_3\left((1-\beta)\,\lambda + \beta\psi_3\right) \geq 0\,. \tag{S1.21}$$

Substituting from the inequality (S1.20) into the inequality (S1.21) implies that $1-\beta \geq 0$. However, this result and the inequality (S1.20) contradict $\psi_3 > 0$. □

The only equilibrium point of the system (S1.4) which can correspond to endemic disease is given by

$$\begin{aligned}
s_2 &= \frac{\psi_3}{\lambda}\,,\\
a_2 &= \frac{\beta\psi_1 - \psi_2\psi_3}{2\beta\psi_1^2} - \frac{\psi_3}{\lambda\psi_1} + \frac{\sqrt{\lambda}\,\sqrt{\lambda(\beta\psi_1 - \psi_2\psi_3)^2 + 4\beta\psi_1\psi_3(\psi_2\psi_3 - \psi_1)}}{2\beta\lambda\psi_1^2}\,,\\
c_2 &= (\psi_1 - 1)a_2\,.
\end{aligned} \tag{S1.22}$$

The requirement that $s_2 \leq 1$ for this equilibrium point to be in $\mathcal{R}_1$ implies that $\lambda \geq \psi_3$. A necessary and sufficient condition for this disease-endemic equilibrium point to be in $\mathcal{R}$ are given in the following proposition.

**Proposition 1.** *The physical region $\mathcal{R}_1$ of the system (S1.4) contains an equilibrium point with $a > 0$ if and only if*

$$\beta > 1 \quad \textit{and} \quad \lambda > \frac{\beta\psi_3}{\beta - 1}\,, \tag{S1.23}$$

*where $\psi_1 = 1 + \frac{\rho}{\delta}$, $\psi_2 = 1 + \frac{\phi\rho}{\delta}$, $\psi_3 = \frac{\delta(1+\rho)}{\delta+\phi\rho}$, and $\delta \geq 1$.*

*Proof.* Observe that the constant term in equation (S1.17) changes sign at $\psi_3 = \frac{\lambda(\beta-1)}{\beta}$. Therefore, one of the two roots of equation (S1.17) changes sign at this value of $\psi_3$. Lemma 1 implies that it must be the largest root. It follows that equation (S1.17) has a single positive root if and only if the inequality (S1.23) holds.

It remains to show that if there is a positive root, then it must always correspond to an equilibrium point in $\mathcal{R}_1$ by verifying that

$$s_2 + a_2 + c_2 = \frac{\psi_3}{\lambda} + \psi_1 a_2 \leq 1\,, \tag{S1.24}$$

which is equivalent to

$$a_2 \leq \frac{1}{\psi_1} - \frac{\psi_3}{\lambda\psi_1}\,. \tag{S1.25}$$

Equations (S1.22) and the inequality (S1.23) imply that

$$\begin{aligned} a_2 &\leq \frac{\beta\psi_1 - \psi_2\psi_3}{2\beta\psi_1^2} - \frac{\psi_3}{\lambda\psi_1} + \sqrt{(\beta\psi_1 - \psi_2\psi_3)^2 + 4(\beta-1)\psi_1(\psi_2\psi_3 - \psi_1)} \\ &= \frac{\beta\psi_1 - \psi_2\psi_3}{2\beta\psi_1^2} - \frac{\psi_3}{\lambda\psi_1} + \frac{\sqrt{((\beta-2)\psi_1 + \psi_2\psi_3)^2}}{2\beta\psi_1^2} \\ &= \frac{1}{\psi_1} - \frac{\psi_3}{\lambda\psi_1} - \frac{1}{\beta\psi_1}\,. \end{aligned} \tag{S1.26}$$

□

# 3 Stability of the Equilibrium Points

Conditions for each of the three equilibrium points — the risk-free equilibrium point, the risk-endemic equilibrium point, and the disease-endemic equilibrium point — to be stable in the physical region $\mathcal{R}$ are determined in this section. We shall prove local asymptotic stability for each of the three fixed points and global asymptotic stability for the risk-free equilibrium point.

The Jacobian matrix of the system of equation (S1.4) is

$$J(s,a,c) = \\ \begin{bmatrix} \beta - 1 - 2\beta s - (2\beta+\lambda)a - (2\beta+\phi\lambda)c & \beta - 2\beta a - (2\beta+\lambda)s - 2\beta c & \beta - 2\beta c - (2\beta+\phi\lambda)s - 2\beta a \\ \lambda a + \phi\lambda c & \lambda s - (\rho+1) & \phi\lambda s \\ 0 & \rho & -\delta \end{bmatrix}. \tag{S1.27}$$

An equilibrium point is locally asymptotically stable if the real parts of all three eigenvalues of the Jacobian matrix evaluated at the equilibrium point are strictly negative. The equilibrium point is unstable if the real part of any eigenvalue is strictly positive.

## 3.1 Risk-Free Equilibrium Point

The risk-free equilibrium point is the only equilibrium point in the the physical region $\mathcal{R}$ when $\beta < 1$. In this case, it is shown in the following theorem that the risk-free equilibrium point is locally asymptotically stable.

**Theorem 1.** *The trivial equilibrium point* $(s_0, a_0, c_0) = (0,0,0)$ *of the system (S1.4) is locally asymptotically stable if* $0 < \beta \leq 1$ *and unstable if* $\beta > 1$*.*

*Proof.* At the equilibrium point $(s_0, a_0, c_0) = (0,0,0)$, the Jacobian matrix (S1.27) is

$$J(s_0,a_0,c_0) = \begin{bmatrix} \beta-1 & \beta & \beta \\ 0 & -(\rho+1) & 0 \\ 0 & \rho & -\delta \end{bmatrix}. \tag{S1.28}$$

The eigenvalues of $J(s_0, a_0, c_0)$ are

$$\nu_{01} = \beta - 1\,, \quad \nu_{02} = -\rho - 1\,, \quad \text{and} \quad \nu_{03} = -\delta\,, \tag{S1.29}$$

which are all real and strictly negative for $0 \leq \beta < 1$. □

In the following theorem, the LaSalle Invariance Principal [2, Chapt. 2: Corollary 6.5] is used to show global asymptotic stability of the risk-free equilibrium point in the physical region $\mathcal{R}$. This implies that any solution $(g(t), s(t), a(t), c(t))$ of the system of equation (S1.2) approaches $(1, 0, 0, 0)$ as $t$ goes to infinity, for all initial values in $\mathcal{R}$.

**Theorem 2.** *If $0 < \beta \leq 1$, then the risk-free equilibrium point $(g_0, s_0, a_0, c_0) = (1, 0, 0, 0)$ is globally asymptotically stable in $\mathcal{R}$.*

*Proof.* First, we use the constraint $g + s + a + c = 1$ to eliminate the equation for $c$ in the system (S1.2) and obtain:

$$\begin{aligned} \frac{dg}{dt} &= s + a + \delta(1 - g - s - a) - \beta g(1 - g)\,, \\ \frac{ds}{dt} &= \beta g(1 - g) - \lambda s(a + \phi(1 - g - s - a) - s\,, \\ \frac{da}{dt} &= \lambda s(a + \phi(1 - g - s - a)) - (\rho + 1)a\,. \end{aligned} \tag{S1.30}$$

We define the Lyapunov-LaSalle function

$$L(g, s, a) = \left(1 - \frac{1}{\delta}\right)(g + s + a) - \ln(g) \tag{S1.31}$$

for $(g, s, a)$ in $\mathcal{R}_2 = \{(g, s, a) \mid g \geq 0,\ s \geq 0,\ a \geq 0,\ g + s + a \leq 1\}$. Lyapunov functions of this form are somtimes referred to as Volterra-style Lyapunov functions [3]. A similar Lyapunov-LaSalle function was used to prove global stability for SIR, SIRS, and SIS models in [4]. The function $L$ satisfies

$$\begin{aligned} \frac{dL}{dt} &= \frac{\partial L}{\partial g}\frac{dg}{dt} + \frac{\partial L}{\partial s}\frac{ds}{dt} + \frac{\partial L}{\partial a}\frac{ds}{dt} \\ &= \left(1 - \frac{1}{g}\right)\big(s + a + \delta(1 - g - s - a)\big) + (\beta - 1)(1 - g) + \left(\frac{1}{\delta} - 1\right)\rho a \\ &\leq 0 \end{aligned} \tag{S1.32}$$

and $\frac{dL}{dt} = 0$ holds only at $(g_0, s_0, a_0)$. Since $\mathcal{R}_2$ is a compact positively invariant set, it follows from the LaSalle Invariance Principal [2, Chapt. 2: Corollary 6.5] that $(1, 0, 0)$ is globally asymptotically stable in $\mathcal{R}_2$ and that $(1, 0, 0, 0)$ is globally asymptotically stable in $\mathcal{R}$. □

## 3.2 Risk-Endemic Equilibrium Point

As $\beta$ increases through one, the risk-endemic equilibrium point $(s_1, a_1, c_1)$ passes through the risk-free equilibrium point into the physical region $\mathcal{R}$. The following theorem implies that a transcritical bifurcation occurs and the risk-endemic equilibrium point becomes locally asymptotically stable.

**Theorem 3.** *The risk-endemic equilibrium point $(s_1, a_1, c_1) = (1 - \frac{1}{\beta}, 0, 0)$ of the system (S1.4) is in the region $\mathcal{R}_1$ and locally asymptotically stable if $\beta > 1$ and $\lambda < \frac{\beta\delta(\rho+1)}{(\beta-1)(\delta+\phi\rho)}$. If $\lambda > \frac{\beta\delta(\rho+1)}{(\beta-1)(\delta+\phi\rho)}$, then it is unstable.*

*Proof.* The Jacobian matrix (S1.27) evaluated at $(s_1, a_1, c_1) = (1 - \frac{1}{\beta}, 0, 0)$ is

$$J(s_1, a_1, c_1) = \begin{bmatrix} 1 - \beta & 2 - \beta - \frac{\lambda(\beta-1)}{\beta} & 2 - \beta - \frac{\phi\lambda(\beta-1)}{\beta} \\ 0 & \frac{\lambda(\beta-1)}{\beta} - \rho - 1 & \frac{\phi\lambda(\beta-1)}{\beta} \\ 0 & \rho & -\delta \end{bmatrix}. \tag{S1.33}$$

The eigenvalues of $J(s_1, a_1, c_1)$ are

$$\begin{aligned}\nu_{11} &= 1-\beta\,,\\ \nu_{12} &= \frac{1}{2}\left(\frac{\lambda(\beta-1)}{\beta}-\rho-1-\delta+\sqrt{\left(\frac{\lambda(\beta-1)}{\beta}-\rho-1+\delta\right)^2+\frac{4\rho\phi\lambda(\beta-1)}{\beta}}\right)\,,\\ \nu_{13} &= \frac{1}{2}\left(\frac{\lambda(\beta-1)}{\beta}-\rho-1-\delta-\sqrt{\left(\frac{\lambda(\beta-1)}{\beta}-\rho-1+\delta\right)^2+\frac{4\rho\phi\lambda(\beta-1)}{\beta}}\right)\,.\end{aligned} \tag{S1.34}$$

Note that if $\lambda < \frac{\beta\delta(\rho+1)}{(\beta-1)(\delta+\phi\rho)}$, then $\frac{\lambda(\beta-1)}{\beta}-\rho-1-\delta<0$. Therefore,

$$\left(\frac{\lambda(\beta-1)}{\beta}-\rho-1-\delta\right)^2 < \left(\frac{\lambda(\beta-1)}{\beta}-\rho-1+\delta\right)^2+\frac{4\rho\phi\lambda(\beta-1)}{\beta} \tag{S1.35}$$

implies that $\nu_{13}<0$ and $\nu_{12}<0$. It is straightforward to verify that the inequality (S1.35) is equivalent to $\lambda < \frac{\beta\delta(\rho+1)}{\lambda(\beta-1)(\delta+\phi\rho)}$, which establishes both the stability and instability criterion in the theorem. □

### 3.3 Disease-Endemic Equilibrium Point

The disease-endemic equilibrium point passes through the risk-endemic equilibrium point and into the physical region $\mathcal{R}$ when $\lambda$ increases through $\frac{\beta\psi_3}{\beta-1}=\frac{\beta\delta(1+\rho)}{(\beta-1)(\delta+\psi\rho)}$ for $\beta>1$. The following theorem implies that at this point a second transcritical bifurcation occurs and the disease-endemic equilibrium point becomes locally asymptotically stable.

**Theorem 4.** *If $\beta>1$, $\psi_2\psi_3\geq\psi_1$ and $\psi_2\leq\psi_1$, then the disease-endemic equilibrium point $(s_2,a_2,c_2)$ is locally asymptotically stable when $\lambda>\frac{\beta\psi_3}{\beta-1}=\frac{\beta\delta(\rho+1)}{(\beta-1)(\delta+\phi\rho)}$. The condition that $\psi_2\leq\psi_1$ is equivalent to $\phi\leq1$, which means that the infectivity in the latent disease phase is less than or equal to the infectivity in the acute disease phase.*

*Proof.* The Jacobian matrix at the disease-endemic equilibrium point given by the equations (S1.22) is

$$J(s_2,a_2,c_2)= \begin{bmatrix} \beta\frac{\lambda-2\psi_3}{\lambda}-1-(\lambda\psi_2+2\beta\psi_1)\,a_2 & \beta\frac{\lambda-2\psi_3}{\lambda}-\psi_3-2\beta\psi_1 a_2 & \beta\frac{\lambda-2\psi_3}{\lambda}-\frac{\psi_3(\psi_2-1)}{\psi_1-1}-2\beta\psi_1 a_2\\ \lambda\psi_2 a_2 & \psi_3-\psi_2\psi_3 & \frac{\psi_3(\psi_2-1)}{\psi_1-1}\\ 0 & \psi_2\psi_3-1 & -\frac{\psi_2\psi_3-1}{\psi_1-1}\end{bmatrix}. \tag{S1.36}$$

The characteristic polynomial of $J_2=J(s_2,a_2,c_2)$ is

$$\nu^3-\mathrm{tr}(J_2)\,\nu^2+\sum_{i=1}^{3}M_i(J_2)\,\nu-\det(J_2)=0\,, \tag{S1.37}$$

where $M_i(J_2)$ are the principal minors of $J_2$. It follows from the Routh-Hurwitz Criterion [5] that all three roots of (S1.37) have strictly negative real parts if and only if

$$\mathrm{tr}(J_2)<0 \tag{S1.38}$$

and

$$\text{tr}(J_2)\sum_{i=1}^{3} M_i(J_2) < \det(J_2) < 0\,. \tag{S1.39}$$

First, we shall show that $\text{tr}(J_2) < 0$. From the matrix (S1.36),

$$\begin{aligned}
\text{tr}(J_2) &= \frac{\beta(\lambda - 2\psi_3)}{\lambda} - 1 - (\lambda\psi_2 + 2\beta\psi_1)a_2 + \psi_3 - \psi_2\psi_3 - \frac{\psi_2\psi_3 - 1}{\psi_1 - 1} \\
&< \frac{\beta(\lambda - 2\psi_3)}{\lambda} - 1 - (\lambda\psi_2 + 2\beta\psi_1)a_2 \\
&= \frac{2\beta\psi_1\left((\beta-1) + \psi_2\psi_3\right) - (\lambda\psi_2 + 2\beta\psi_1)\left(\beta\psi_1 - \psi_2\psi_3\right)}{2\beta\psi_1^2} \\
&\quad - \frac{(\lambda\psi_2 + 2\beta\psi_1)\sqrt{\lambda}\sqrt{\lambda(\beta\psi_1 - \psi_2\psi_3)^2 + 4\beta\psi_1\psi_3(\psi_2\psi_3 - \psi_1)}}{2\beta\lambda\psi_1^2}\,,
\end{aligned} \tag{S1.40}$$

because $\psi_3 - \psi_2\psi_3 - \frac{\psi_2\psi_3 - \psi_1}{\psi_1 - 1} < 0$. Squaring terms in the last equation of (S1.40) gives that $\text{tr}(J_2) < 0$ if

$$\begin{aligned}
X &= \lambda^2\Big(2\beta\psi_1\left((\beta-1)\psi_1 + \psi_2\psi_3\right) - (\lambda\psi_2 + 2\beta\psi_1)\left(\beta\psi_1 - \psi_2\psi_3\right)\Big)^2 \\
&\quad - \left((\lambda\psi_2 + 2\beta\psi_1)\sqrt{\lambda}\sqrt{\lambda\left(\beta\psi_1 - \psi_2\psi_3\right)^2 + 4\beta\psi_1\psi_3\left(\psi_2\psi_3 - \psi_1\right)}\right)^2 \\
&< 0\,.
\end{aligned} \tag{S1.41}$$

Simplifying $X$, we have that

$$X = 4\beta\lambda\psi_1 Q(\lambda)\,, \tag{S1.42}$$

where

$$Q(\lambda) = -\lambda^2\beta(\beta-1)\psi_1^2\psi_2 + \lambda(\beta(1-\beta^2)\psi_1^3 + 2\beta^2\psi_2\psi_3 - \beta\psi\psi_2^2\psi_3^2) - 4\beta^2\psi_1^2\psi_3(\psi_2\psi_3 - \psi_1)\,. \tag{S1.43}$$

We need to show that $Q(\lambda) < 0$ when $\lambda > \frac{\beta\psi_3}{\beta-1}$. Evaluating $Q(\lambda)$ at $\lambda = \frac{\beta\psi_3}{\beta-1}$ gives

$$Q\left(\frac{\beta\psi_3}{\beta-1}\right) = -\frac{\beta^2\psi_1\psi_3}{\beta-1}\left(\psi_2^2\psi_3^2 + (3\beta-4)\psi_1\psi_2\psi_3 + (\beta^2 - 4\beta + 3)\psi_1^2\right). \tag{S1.44}$$

The term inside the brackets on the right side of equation (S1.44) is a quadratic polynomial in $\psi_2\psi_3$. Rescaling this polynomial by $\psi_1$, we have that $Q\left(\frac{\beta\psi_3}{\beta-1}\right) < 0$, because

$$p(x) = x^2 + (3\beta-4)x + \beta^2 - 4\beta + 3 > 0 \tag{S1.45}$$

for $x \geq 1$ and $\beta > 1$. The maximum value of $Q(\lambda)$ occurs at

$$\lambda = \frac{2\beta\psi_1\psi_2\psi_3 - (\beta^2-1)\psi_1^2 - \psi_2^2\psi_3^2}{2(\beta-1)\psi_1\psi_2} < \frac{\beta\psi_3}{\beta-1}\,. \tag{S1.46}$$

Therefore, $Q\left(\frac{\beta\psi_3}{\beta-1}\right) < 0$ and $Q(\lambda)$ is a decreasing function of $\lambda$ for $\lambda > \frac{\beta\psi_3}{\beta-1}$. This establishes that $\text{tr}(J_2) < 0$.

Next, we show that $\det(J_2) < 0$. Note that the radical in the expression for $a_2$ in equations (S1.22) is strictly positive, because $\beta > 1$. This gives the bound

$$\begin{aligned}\det(J_2) &= \frac{\lambda\psi_2 a_2(\psi_2\psi_3)}{\psi_1 - 1}\left(\beta\psi_1\left(1 - \frac{2\psi_3}{\lambda} - 2\psi_1 a_2\right) - \psi_2\psi_3\right) \\ &< \frac{\lambda\psi_2 a_2(\psi_2\psi_3)}{\psi_1 - 1}\left(\beta\psi_1\left(1 - \frac{2\psi_3}{\lambda} - 2\psi_1\left(\frac{\beta\psi_1 - \psi_2\psi_3}{2\beta\psi_1^2} - \frac{\psi_3}{\lambda\psi_1}\right)\right) - \psi_2\psi_3\right) \\ &= 0\,.\end{aligned} \tag{S1.47}$$

Finally, we show that $\operatorname{tr}(J_2)\sum_{i=1}^{3} M_i(J_2) < \det(J_2)$. Note that

$$\operatorname{tr}(J_2)\sum_{i=1}^{3} M_i - \det(J_2) = D_1(M_2 + M_3) + D_2(M_1 + M_3) + D_3(M_1 + M_2)\,, \tag{S1.48}$$

where we have simplified the notation by writing $M_i$ for $M_i(J_2)$ and $D_i$ is the diagonal element $(J_2)_{ii}$. The principal minors of $J_2$ are

$$\begin{aligned}M_1 &= 0 \\ M_2 &= -\left(\frac{\beta(\lambda - 2\psi_3)}{\lambda} - 1 - (\lambda\psi_2 + 2\beta\psi_1 a_2)\right)\left(\frac{\psi_2\psi_3 - 1}{\psi_1 - 1}\right) \\ M_3 &= \left(\frac{\beta(\lambda - 2\psi_3)}{\lambda} - 1 - (\lambda\psi_2 + 2\beta\psi_2)\right)(\psi_3 - \psi_2\psi_3) - \left(\frac{\beta(\lambda - 2\psi_3)}{\lambda} - \psi_3 - 2\beta\psi_1 a_2\right)\lambda\psi_2 a_2\,.\end{aligned} \tag{S1.49}$$

Substituting this into the expression (S1.48) gives

$$\begin{aligned}&\operatorname{tr}(J_2)\sum_{i=1}^{3} M_i - \det(J_2) \\ &\quad= \left(\frac{\beta(\lambda - 2\psi_3)}{\lambda} - 1 - (\lambda\psi_2 + 2\beta\psi_1)a_2\right)^2\left(\psi_3 - \psi_2\psi_3 - \frac{\psi_2\psi_3 - 1}{\psi_1 - 1}\right) \\ &\qquad - \left(\frac{\beta(\lambda - 2\psi_3)}{\lambda} - 1 - (\lambda\psi_2 + 2\beta\psi_1)a_2\right)\left(\frac{\beta(\lambda - 2\psi_3)}{\lambda} - \psi_3 - 2\beta\psi_1 a_2\right)\lambda\psi_2 a_2 \\ &\qquad + \left(\frac{\beta(\lambda - 2\psi_3)}{\lambda} - 1 - (\lambda\psi_2 + 2\beta\psi_1)a_2\right)(\psi_3 - \psi_2\psi_3)^2 \\ &\qquad - \left(\frac{\beta(\lambda - 2\psi_3)}{\lambda} - \psi_3 - 2\beta\psi_1 a_2\right)(\psi_3 - \psi_2\psi_3)\,\lambda\psi_2 a_2 \\ &\qquad + \left(\frac{\beta(\lambda - 2\psi_3)}{\lambda} - 1 - (\lambda\psi_2 + 2\beta\psi_1)a_2\right)\left(\frac{\psi_2\psi_3 - 1}{\psi_1 - 1}\right)^2\,.\end{aligned} \tag{S1.50}$$

It follows from Proposition 1, that if $\lambda > \frac{\beta\psi_3}{\beta-1}$, then $a_2 > 0$. Furthermore, when proving that $\operatorname{tr}(J_2) < 0$ above, we showed that $\lambda > \frac{\beta\psi_3}{1-\beta}$ implies that

$$\frac{\beta(\lambda - 2\psi_3)}{\lambda} - 1 - (\lambda\psi_2 + 2\beta\psi_1)a_2 < 0\,. \tag{S1.51}$$

Therefore, $Y = \frac{\beta(\lambda-2\psi_3)}{\lambda} - \psi_3 - 2\beta\psi_1 a_2$ is the only expression in (S1.50) whose sign is unknown and it

remains only to show that $Y \leq 0$. Substituting for $a_2$ into $Y$ gives the bound

$$\begin{aligned} Y &< \frac{\beta(\lambda - 2\psi_3)}{\lambda} - \psi_3 - 2\beta\psi_1 \left( \frac{\beta\psi_1 - \psi_2\psi_3}{2\beta\psi_1^2} - \frac{\psi_3}{\lambda\psi_1} \right) \\ &= -\psi_3 + \frac{\psi_2\psi_3}{\psi_1} \\ &\leq 0\,, \end{aligned} \tag{S1.52}$$

because $\psi_2 \leq \psi_1$. □

# References


1. Meiss JD (2007) Differential Dynamical Systems. Philadelphia: SIAM.
2. LaSalle JP (1976) The Stability of Dynamical Systems. Philadelphia: SIAM.
3. Fall A, Iggidr A, Sallet G, Tewa JJ (2007) Epidemiological models and Lyapunov functions. Mathematical Modelling of Natural Phenomena 2: 62–83.
4. Korobeinikov A, Wake GC (2002) Lyapunov functions and global stability for SIR, SIRS, and SIS epidemiological models. Applied Mathematics Letters 15: 955–960.
5. Gantmacher FR (2005) Applications of the Theory of Matrices. Mineola, N. Y.: Dover.
